# Infective Endocarditis Complicated by Carotid Artery Thrombosis and Infectious Intracranial Aneurysm: A Case Report

**DOI:** 10.7759/cureus.97607

**Published:** 2025-11-23

**Authors:** Rui Li, Min Feng

**Affiliations:** 1 Neurology, Chongqing Qianjiang Central Hospital, Chongqing, CHN; 2 Pediatrics, Chongqing Qianjiang Central Hospital, Chongqing, CHN

**Keywords:** carotid artery thrombosis, cerebral venous infarction, infectious intracranial aneurysm, infective endocarditis, intracerebral hemorrhage

## Abstract

Infective endocarditis concurrently complicated by carotid artery thrombosis and rupture of an intracranial aneurysm with hemorrhage is clinically rare. Its treatment presents contradictions, as both the carotid artery thrombosis and the rupture of the intracranial aneurysm with hemorrhage need to be considered, posing challenges to clinical management. This report describes a patient who presented with hemiplegia. During the physical examination, we found that the patient had a fever of 38.5°C and a systolic murmur was auscultated in the mitral valve area. Subsequently, blood cultures and echocardiography were performed, leading to a diagnosis of infective endocarditis. A brief discussion on the neurological complications of infective endocarditis is also provided. Cranial vascular examinations revealed carotid artery thrombosis and intracranial aneurysms, and subsequent consideration was given to aneurysmal rupture with hemorrhage. After anti-infective treatment, the patient's symptoms improved. Through this case report, we emphasize that when patients present with neurological complications accompanied by fever and cardiac murmurs, infective endocarditis should be considered as a potential diagnosis.

## Introduction

The clinical manifestations of infective endocarditis vary significantly and may present as acute, subacute, or chronic symptoms, depending on the type of pathogenic microorganism, underlying cardiac disease, and comorbidities. Approximately 90% of patients exhibit symptoms such as fever, night sweats, fatigue, weight loss, and anorexia. About 85% of patients have a heart murmur or a newly developed murmur, with approximately 25% presenting with embolic signs at the time of presentation [1,2]. Positive blood cultures remain the cornerstone of infective endocarditis diagnosis and provide viable organisms for identification and antimicrobial susceptibility testing. In this case, the patient was admitted to our hospital's Neurology Outpatient Department due to hemiplegia. Upon examination, the patient was found to have fever, fatigue, and a heart murmur. Blood cultures were performed, and the diagnosis of infective endocarditis was made.

## Case presentation

**Figure 1:**
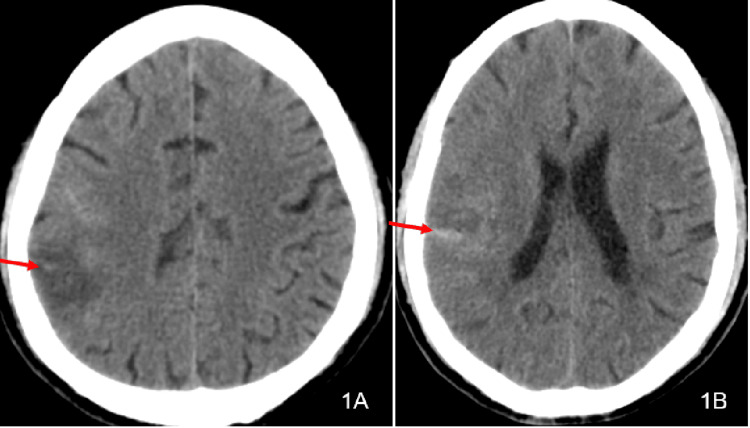
(A-B) Cerebral computed tomography on June 1 indicating right parietal lobe cerebral infarction with minor hemorrhage (arrow indicated)

**Figure 2:**
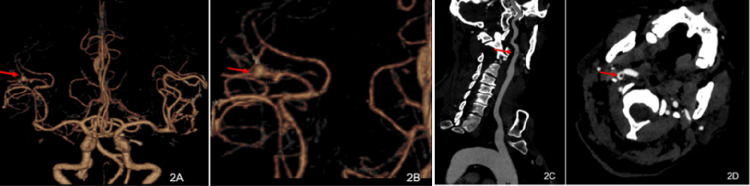
(A-B) Head and neck computed tomography angiography on June 1 indicating multiple aneurysms in the M3 segment of the right middle cerebral artery (arrow indicated). (C-D) Head and neck computed tomography angiography on June 1 indicating mural thrombus formation in the C1 segment of the right internal carotid artery (arrow indicated)

A 54-year-old, unmarried man was admitted to our hospital on June 1, 2025, escorted by his younger brother, due to left-sided limb weakness for 15 hours. Fifteen hours prior to admission, the patient developed left-sided limb weakness without obvious precipitating factors. He had difficulty holding objects in the left upper limb but was still able to lift it. Moreover, he had difficulty standing with his left lower limb, making independent walking impossible. His symptoms were accompanied by fever (approximately 38.5°C). As regards his past medical history, he had no history of hypertension, diabetes mellitus, coronary heart disease, valvular heart disease, infectious diseases, etc. In the physical examination, his temperature was 38.8°C, pulse rate 124 beats per minute, respiratory rate 22 breaths per minute, and blood pressure 129/88 mmHg. The patient was conscious, speech was slightly unclear, and cooperation with the examination was good. Meningeal irritation signs were negative (-). Both pupils were equal in size and round, with a diameter of 3 mm. Direct and indirect light reflexes were brisk, and the accommodation reflex was normal. The left nasolabial fold was shallow, and the tongue protruded to the left. Left superficial sensation was worse than the right. Bilateral lung breath sounds were coarse, with no dry or wet rales heard. Arrhythmia was present, and a systolic blowing murmur could be auscultated at the apex of the heart. The abdomen was soft without tenderness. Limb muscle tone was normal. Right muscle strength was grade V, and left limb muscle strength was grade III. The National Institutes of Health Stroke Scale (NIHSS) score was 8 points (facial palsy 2 points, left upper limb 2 points, left lower limb 2 points, sensation 1 point, dysarthria 1 point). The head computed tomography (CT) suggested right parietal lobe cerebral infarction with minor hemorrhage (Figure 1A-1B). Computed tomography angiography (CTA) showed mural thrombus formation in the C1 segment of the right internal carotid artery (Figure 2C-2D), stenosis of the M2 segment of the right middle cerebral artery, and multiple aneurysms in the M3 segment of the right middle cerebral artery (Figure 2A-2B). Bedside cardiac ultrasound revealed the following: left atrial and left ventricular enlargement, left ventricular systolic function within the lower normal range, mid-atrial septum protruding into the right atrium, and high echogenicity on the mitral valve possibly indicating vegetation, with a size of 7.6×4.7 mm. 

**Figure 3:**
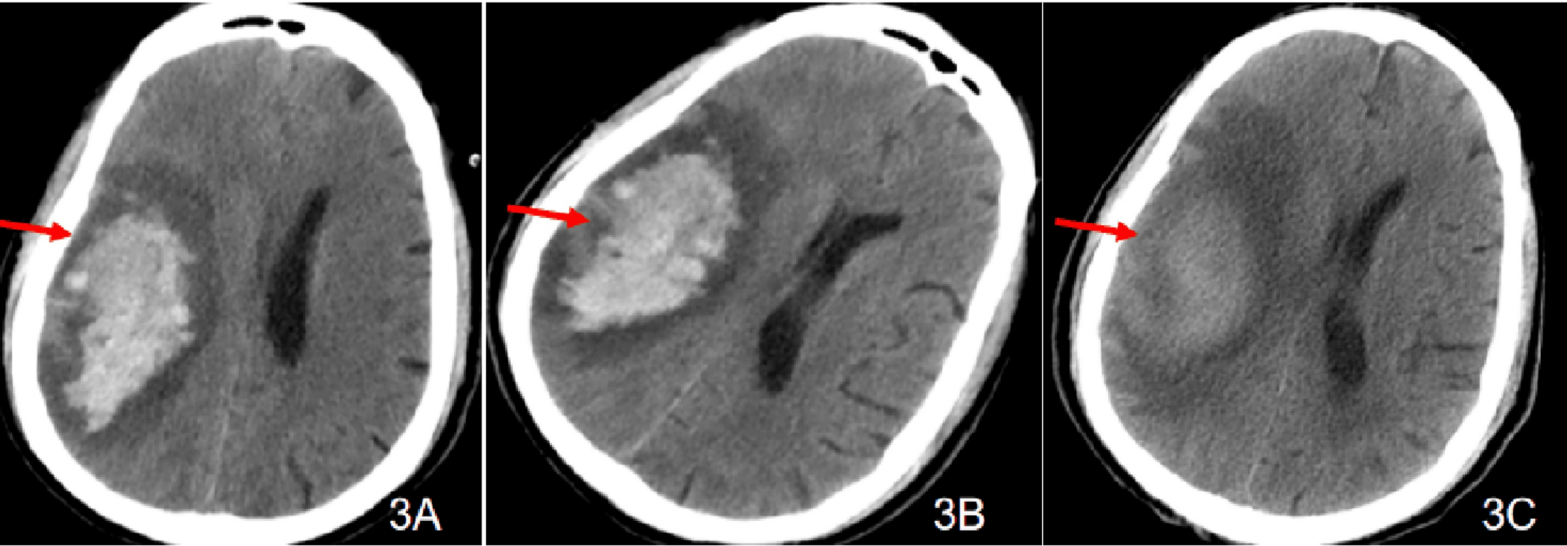
(A-B) Head computed tomography review on June 3 indicating a significant increase in the right parietal lobe hemorrhage (arrow indicated). (C) Head computed tomography review on June 16 indicating the absorption of the right parietal lobe hemorrhage (arrow indicated)

Subsequently, blood culture identified *Streptococcus *in the blood. A cardiology consultation was requested, leading to a diagnosis of subacute infective endocarditis with infective mitral valve vegetation. The patient was treated with antipyretic therapy using piperacillin-tazobactam and vancomycin. Due to carotid artery thrombosis complicated by cerebral hemorrhage, anticoagulant therapy was not administered. On June 3 at 19:05, the symptoms worsened with impaired consciousness, shallow coma, and aggravated limb weakness. Review of the head CT showed significantly increased intracranial hemorrhage with obvious mass effect (Figure 3A-3B). The patient was transferred to the intensive care unit (ICU) for treatment, including endotracheal intubation, sedation, ventilator-assisted ventilation, and the discontinuation of butylphthalide. Blood cultures on June 1 and June 2 were positive, indicating *Streptococcus* bacteremia. Antimicrobial therapy with piperacillin-tazobactam combined with vancomycin was administered, along with dehydration with mannitol and nutritional support. The patient was transferred out of the ICU on June 11. Review of the cranial CT on June 16 showed significant absorption of the hemorrhage (Figure 3C). Blood culture results on June 14 and June 19 were negative. On June 19, the patient requested discharge. Vital signs at discharge were as follows: temperature 36.8℃, pulse rate 124 beats per minute, respiratory rate 22 breaths per minute, and blood pressure 115/81 mmHg. The patient was in a drowsy state with slurred speech, a systolic blowing murmur was auscultated at the apex of the heart, and left limb muscle strength was grade 0. Due to family reasons, the patient did not return to the hospital for clinical follow-up. A telephone follow-up after three months showed clear consciousness, clear speech, left limb muscle strength at grade IV, and the ability to walk independently. The modified Rankin Scale (MRS) score was 2 points.

The patient's laboratory test results at the time of initial admission are shown in Table 1.

**Table 1:** Laboratory values CRP: C-reactive protein; PT: prothrombin time; INR: international normalized ratio; APTT: activated partial thromboplastin time; NT-ProBNP: N-terminal pro-brain natriuretic peptide

Parameter	Patient value	Reference range
White blood cells	10.7*×*10^9^/L	3.5-9.5*×*10^9^/L
Red blood cells	2.9*×*10^12^/L	4.3-5.8*×*10^12^/L
Hemoglobin	79 g/L	130-175 g/L
Neutrophil ratio	77.5%	40-75%
CRP	103.36 mg/L	0-10 mg/L
PT	16.1 s	11-14.5 s
INR	1.32	0.85-1.15
APTT	46.6 s	28-44 s
Fibrinogen	4.61 g/L	2-4 g/L
Serum albumin	29.1 g/L	40-55 g/L
Procalcitonin	0.62 ng/ml	0-0.05 ng/ml
NT-ProBNP	4083 pg/ml	0-125 pg/ml

## Discussion

Patients with infective endocarditis are prone to hemorrhagic and embolic strokes, with the latter being the most common [3,4]. Risk factors for embolic infarction include patient-related factors such as advanced age, atrial fibrillation, prior embolic events, multiple-valve endocarditis, mitral valve vegetations, and larger vegetations [5,6]. Microbial pathogens that primarily contribute to increased embolism risk are *Staphylococcus aureus* and *Candida *species [7,8]. Embolic events occur in 20-40% of infective endocarditis patients and are associated with increased morbidity and mortality [9]. Even after the initiation of antibiotic therapy, systemic embolism risk remains highest during the initial phase of infection and gradually decreases after two weeks [10]. Systemic embolism occurs in 20-50% of cases due to left ventricular vegetations. Cerebral embolism is a major cause of death in infective endocarditis, with more than 40% of cases typically involving the middle cerebral artery [11]. Unlike other cardioembolic strokes, anticoagulant therapy cannot reduce the embolism risk in patients with infective endocarditis and may even increase the risk of intracranial hemorrhage. After appropriate antibiotic treatment, the incidence of severe embolic events also significantly decreases [12]. In this case, the patient was found to have a mural thrombus in the C1 segment of the right internal carotid artery upon presentation, along with a right parietal lobe infarction. It was considered that partial thrombus embolization led to cortical infarction in the right parietal lobe. Due to concurrent minor bleeding, anticoagulation and mechanical thrombectomy were not performed. The patient received anti-infective treatment, and no new embolic events were observed during hospitalization. Unfortunately, due to personal and family reasons, the patient did not undergo follow-up vascular imaging to assess the status of the carotid artery thrombus.

Cerebral thromboembolism is an indication for heart valve surgery, especially when accompanied by heart failure or other infectious complications. One of the contraindications for early cardiac valve surgery is the presence of intracranial hemorrhage. For patients with cerebral hemorrhage combined with cerebral infarction, cardiac surgery should be delayed for at least four weeks [10]. Infectious intracranial aneurysms or mycotic aneurysms are rare complications of systemic or central nervous system infection in infective endocarditis patients, accounting for 2-9% of infective endocarditis patients according to literature reports [13,14]. Due to damage to the elastic fiber layer of the arterial wall, the intracranial internal carotid artery aneurysm is more prone to rupture and bleeding. Moreover, the bleeding risk of infectious intracranial aneurysm is unrelated to its location and aneurysm diameter, but only associated with morphology: saccular infectious intracranial aneurysm has a significantly higher rupture risk than fusiform infectious intracranial aneurysm [15]. The treatment options for infectious cerebral aneurysms include antibiotic therapy (which may be combined with endovascular treatment or surgical treatment), but the existing evidence mainly comes from case reports and retrospective studies [16]. For large infectious aneurysms, neurosurgical surgery or endovascular treatment is recommended [10]. In this case, the patient had indications for heart valve surgery and concurrent bleeding, so cardiac surgery should be delayed by at least four weeks. Additionally, we identified multiple aneurysms in the M3 segment of the right middle cerebral artery. After evaluation by the departments of cardiology, thoracic surgery, and neurosurgery, antibiotic therapy was selected. Three days later, the patient's symptoms worsened, with an intracranial hematoma appearing, suggesting possible aneurysm rupture and bleeding. After treatment, the hematoma was absorbed, and the patient was discharged. A telephone follow-up was conducted three months after discharge; the patient was conscious, spoke clearly, had left limb muscle strength at grade IV, and could walk independently.

## Conclusions

When presenting with stroke and concurrent fever and heart murmur, the possibility of stroke caused by infective endocarditis should be highly suspected. Early diagnosis and appropriate antibiotic treatment are of great significance for reducing complications related to infective endocarditis and lowering mortality. Cases of infective endocarditis complicated with carotid artery thrombosis and rupture of an infectious intracranial aneurysm with hemorrhage are clinically rare. The diagnosis and treatment of infective endocarditis still face challenges due to its diverse clinical manifestations, making multidisciplinary team collaboration crucial.
